# Comparative study of effect of *Akkermansia muciniphila* and its extracellular vesicles on toll-like receptors and tight junction 

**Published:** 2019

**Authors:** Fatemeh Ashrafian, Ava Behrouzi, Arefeh shahriary, Sara Ahmadi Badi, Mehdi Davari, Shohre khatami, Fatemeh Rahimi Jamnani, Abolfazl Fateh, Farzam Vaziri, Seyed Davar Siadat

**Affiliations:** 1 *Department of Mycobacteriology and Pulmonary Research, Pasteur Institute of Iran, Tehran, Iran*; 2 *Microbiology Research Centre, Pasteur Institute of Iran, Tehran, Iran*; 3 *Department of Biochemistry, Pasteur Institute of Iran, Tehran, Iran *

**Keywords:** Gut microbiota, Akkermansia muciniphila, Toll-like receptor, Tight junction protein, Extracellular vesicle

## Abstract

**Aim:**

We assessed effect of *Akkermansia muciniphila* and its extracellular vesicles on toll-like receptors and tight junction expression in human epithelial colorectal adenocarcinoma cells (Caco-2).

**Background:**

The intestinal microbiota plays an important role in the intestinal homeostasis through its metabolites and derivatives. Interacting with immune cells and intestinal epithelial pattern recognition receptors (PRRs), intestinal microbiota regulates the function of the digestive barrier and inflammation caused by the metabolic diseases.

**Methods:**

*A. muciniphila* was cultured on a mucin-containing medium and its EVs was extracted by ultracentrifugation. This bacterium was treated in the MOI=10 and its EVs at the concentrations of 0.1, 0.5 and 5 µg on Caco-2 cells. After 24 hours, the expression of tight junction and toll-like receptor genes were investigated by quantitative real time PCR method.

**Results:**

*A. muciniphila* increased the expression of *tlr2 *and* tlr4*. However, EVs at all of the concentrations showed a decrease in *tlr4* expression. EVs at the concentrations of 0.1 and 0.5 µg/ml decreased the expression of *tlr2*.* A. muciniphila* significantly increased the expression of *ocldn and cldn4*. Both this bacterium and EVs increased the expression of *zo2* in the cell line. Furthermore, this data show that *A. muciniphila* derived EVs have a dose-independent effect on Caco-2 cells.

**Conclusion:**

This preliminary research shows* A. muciniphila* and its EVs both may increase the integrity of the intestinal barrier. *A. muciniphila* derived EVs also reduces the inflammation so that EVs of this bacterium can be used as an appropriate target for the treatment of metabolic syndrome and inflammatory bowel diseases.

## Introduction

 The intestinal microbiota is the largest human microbiota reservoir that plays a significant role in human health and is involved in regulating many host physiological pathways such as the regulation of the digestive system stimulation, permeability of the digestive barrier, fat distribution, and energy homeostasis (1, 2). Intestinal microbiota reacts through MAMPs in the outer membrane surface, producing metabolites, enzymes, and secretion of vesicles with toll-like receptors (TLRs), resulting in tolerance and maintenance of intestinal immune homeostasis. Gut microbiota also interacts with the intestinal barrier, preserves the integrity of the intestinal barrier and provides protection against pathogenic bacteria (3, 4).

Gut microbiota dysbiosis can occur due to a variety of reasons, reduces the expression of proteins involved in the formation of tight junctions (TJ) such as zonula occludens and occludin and TLRs, altering the permeability of the gastrointestinal epithelial barrier and creating inflammatory cytokines (5, 6). The reduction of the integrity of the intestinal barrier and the formation of inflammation is associated with the onset and development of many diseases, including inflammatory intestinal diseases and metabolic diseases. Manipulation of the intestinal microbiota through prebiotics and probiotics can increase the beneficial bacteria, reduce inflammation and improve the function of the intestinal barrier (7).

One of the normal flora bacteria in the gastrointestinal tract, which has been recently introduced as the next-generation probiotics candidate, is the *Akkermansia muciniphila (A.muciniphila)* bacterium. *A. muciniphila *is an anaerobic Gram-negative bacterium belonging to the verrucomicrobia phylum that is located in the gastrointestinal mucosal layer and 1-4% of the fecal microbiota communities in the gut. This bacterium has been identified as a mucin-degrading bacterium and is considered as the closest bacterium to epithelial cells (8, 9). *A. muciniphila *also increases the number of goblet cells and mucus secretion, restoring the thickness of the intestinal mucus caused by High Fat Diet (HFD), thereby increasing the epithelial barrier integrity (2, 10). This bacterium plays an important role in the relationship between the gastrointestinal tract microbiota and the host, controlling the function of the digestive barrier and other physiological and hemostatic functions during obesity and type 2 diabetes (2, 11). Due to its role in stabilizing the thickness of the intestinal mucosa and maintaining the integrity of the digestive barrier, it increases the colonization of beneficial bacteria and reduces pathogenic bacteria (12). 

Like other bacteria, gastrointestinal tract commensal bacteria produce extracellular vesicles (EVs). EVs have a spherical shape of 20 to 250 nm in diameter that is produced by many Gram positive and negative bacteria. These molecules are released and secreted at all stages of bacterial growth and in many culture media and different stages of bacterial life during pathogenicity and as a normal flora (13). EVs of gastrointestinal commensal bacteria play a role in adjusting the immune system and regulating the intestinal hemostasis and the epithelial intestinal permeability (14). In a recent study, it has been found that *A. muciniphila *derived EVs (*A.m*EV) has a better effect on the regulation of the immune system and intestinal hemostasis and protection of colitis in mice than the bacterium itself (15). Given that the intestine is a place for interaction between microbiota and host and Caco-2 cell line represents intestinal cells and is the best model for testing TJ proteins (16, 17), we have decided to investigate the effect of the bacterium and its EVs on this cell line. Our aim has been to compare the effects of *A. muciniphila *and its EVs on inflammation and TJ *in vitro*. 

## Methods

**Culture of** ***A. muciniphila***

*A. muciniphila *Muc^T ^(ATCC BAA-835) was purchased from DSMZ institute. It was cultured anaerobically in a basal mucin-based medium under the anaerobic conditions (80% N_2_, 10% H_2 _and 10% CO_2_) at 37 °C for 3-7 days (9). After growth, *A. muciniphila *was inoculated to brain heart infusion broth (BHI) (Quelab) supplemented with 0.5% mucin (Sigma-Aldrich) under the above conditions for 24 hours. Cultures were centrifuged and washed with an anaerobic PBS and used for EVs extraction. 

**Table 1:** Sequence of primers used in qPCR

Primer name	Forward primer	Reverse primer	Product size (bp)
*gapdh*	GGAGCGAGATCCCTCCAAAAT	GGCTGTTGTCATACTTCTCATGG	197
*tlr*_2_	TTATCCAGCACACGAATACACAG	AGGCATCTGGTAGAGTCATCAA	160
*tlr*_4_	AGACCTGTCCCTGAACCCTAT	CGATGGACTTCTAAACCAGCCA	147
*zo1*	CAACATACAGTGACGCTTCACA	CACTATTGACGTTTCCCCACTC	105
*zo2*	ATGGAAGAGCTGATATGGGAACA	TGCTGAACTGCAAACGAATGAA	242
*zo3*	CAGACAGGCGACCACATCG	GGTGCAGGTCTTGAGTATCTGA	90
*ocldn*	AAGAGTTGACAGTCCCATGGCATAC	ATCCACAGGCGAAGTTAATGGAAG	133
*cldn4 *	GGGGCAAGTGTACCAACTG	GACACCGGCACTATCACCA	109

**EV**s** Extraction**

Bacterial cultures were pelleted at 10000 g for 20 min. The EVs were extracted with ultracentrifuge at 150000g for 2 hours at 4 °C as previously described (18). The pellets were re-suspended in 3% sucrose and stored at − 80 °C. The morphology of EVs was monitored by scanning electron microscope (SEM), then total protein concentration measured by the Bradford Protein Assay. 

**Cell culture Treatment**

The Caco-2 (ATCC® HTB-37) as human epithelial colorectal adenocarcinoma cell line were cultured at 37°C in 5% CO2 in Dulbecco's Modified Eagle's Medium (DMEM)(Gibco), supplemented with 10% heat inactivated fetal bovine serum (FBS)(Gibco) and 1% penicillin-streptomycin (Gibco) in six well plates (Sorfa). Afterward, Caco2 were infected with *A. muciniphila *at Multiplicity of infection (MOI) 10 ratios (i.e. 10 bacteria per cell). Besides, different concentrations of EVs (0.1, 0.5 and 5 µg) were used for treatment of Caco-2 cell culture. For each experiment, equal volumes of PBS or sucrose were used as control separately. All of the experiments were performed in triplicate.

**Trypan blue exclusion assay**

 The effects of *A. muciniphila *and its EVs on the viability of cells were determined using trypan blue exclusion assay as described. Briefly, cells were treated with MOI _10_ *A. muciniphila *and variable concentrations of EVs for 24 h at 37 °C. Treated cells incubated and then trypsinized. Equal volume of trypan blue mixed with cell suspension and calculated using a hemocytometer. Live and dead cells were counted with confocal microscopy. The viability percentage was determined using the following formula: 

% Viability= (Live Cell Count/Total Cell Count) ×100

**RNA extraction and cDNA synthesis**

After 24 h post infection, total RNA was extracted from Caco-2 cells using RNeasy Plus Mini kit (Qiagen). cDNA was synthesis from 500 ng total RNA using PrimeScript RT Reagent Kit (Takara) according to the manufacture's instruction. 

**Quantitative Real-Time PCR Experiments**

Real-time PCR was performed by 2X SYBR Premix Ex Taq II (Takara) with Roche LightCycler 96 instrument. The mRNA expression levels of *tlr*_2_*, tlr*_4, _*ocldn, zo1*_,_* zo2*_,_* zo3* and *cldn4 *were evaluated. *gapdh* was used as a reference gene. Finally, raw data were analyzed according to the ΔΔCT method and *gapdh* was used as an internal control to normalize gene expression. Sequence of primers used in this study was prepared from the Primer Bank (Table 1). All of the experiments were performed in triplicate. 

**Statistical analysis**

Analysis of relative gene expression data was performed by GraphPad Prism 6 (GraphPad, La Jolla, CA). Data comparisons was performed with unpaired t-test. Results with P<0.05 were considered to be statistically significant.

## Results

**Morphology and characterization of EV**s 

**Figure 1:**
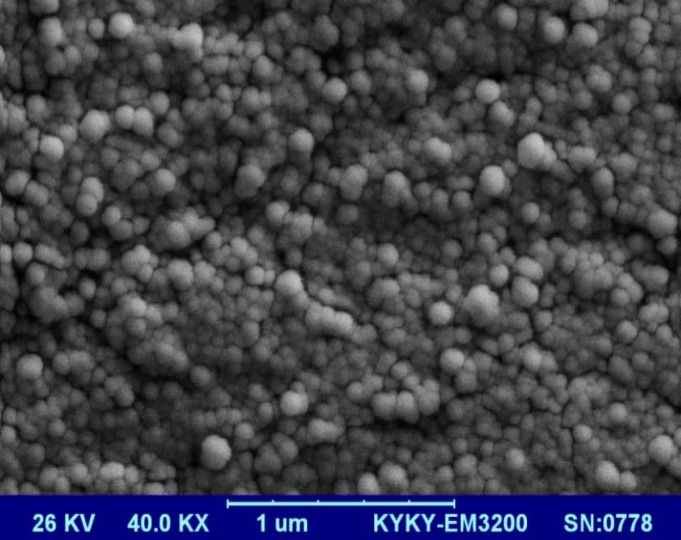
A Scanning electron microscopy of *A.m*EVs (magnification: ×40K)

 Scanning Electron Microscopy (SEM) analysis of *A.m*EV showed a spherical shape and a range of 40 to 150 nm in size (Figure 1). 

**Effects of** ***A. muciniphila*** **and its EV**s** on TLRs**

After 24 hours, no difference in cell viability was observed in both control and treatment groups. It is widely approved that, the gut flora influences the intestinal immune system and have a crucial role in the immune system homeostasis by interaction with TLRs. Thus, to determine the effect of *A. muciniphila *and *A.m*EV in Caco2 Cell line, this bacterium and its EVs concentrations treated overnight and then gene expressions were measured by qPCR. 

**Figure 2:**
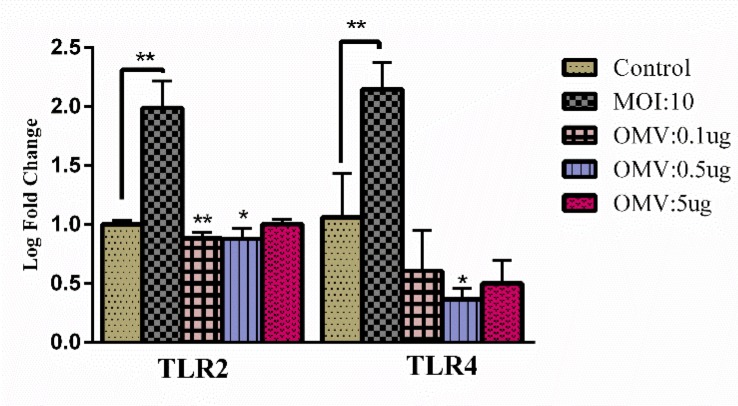
Effects of *A. muciniphila *and its EVs on TLRs. The Caco-2 cells were treated with *A. muciniphila *at MOI 10 and different concentrations of *A.m*EV (0.1, 0.5 and 5 µg); *, **'P<0.05 and P<0.01 were considered statistically significant, respectively. *gapdh* was used as an internal control

*A. muciniphila *in the Caco2 cell line has significantly increased *tlr2* and *tlr4* gene expression. In response to EVs 0.1 and 0.5 µg/ml, expression of *tlr2* were decreased but not in 5 µg/ml. Interestingly, the expression of *tlr4* was significantly decreased in response to 0.5 µg/ml of EVs concentrations (Figure 2).

**Figure 3:**
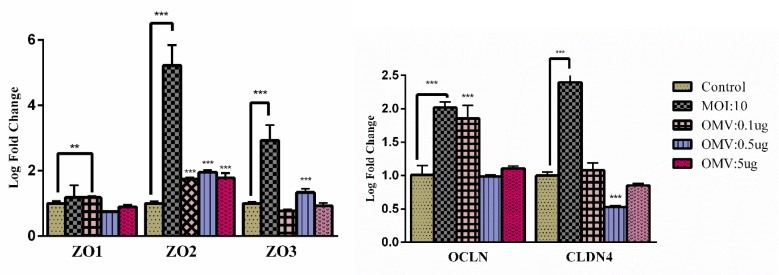
Effects of *A. muciniphila *and its EV on tight junctions: The Caco-2 cells were treated with *A. muciniphila *at MOI 10 and different concentrations of *A.m*EV (0.1, 0.5 and 5 µg); **, ***; P<0.01 and P<0.001 were considered statistically significant, respectively. *gapdh* was used as an internal control

**Effects of** ***A. muciniphila*** **and its EV**s** on tight junctions**

Gut microbiota and their derivatives increases TJ proteins in the intestinal barrier, improves intestinal homeostasis and thereby create healthy conditions in the gut. Significantly* A. muciniphila *increased TJ gene expressions such as *ocldn*, *cldn4*, *zo2* and *zo3* but a slightly increased expression of *zo1*.

*A.m*EV increased expression of *ocldn *(Figure 3A) and* zo1* at the mRNA level in response to 0.1 µg/ml concentration but decreased *zo3 *expression (not significant). At the EVs 0.5 µg/ml expression of *zo3 *was increased. In response to EVs 0.5 µg/ml expression of *cldn4* was decreased. All of the EVs concentrations significantly increased *zo2 *expression. (Figure 3B). This result initially shows that the effect of *A.m*EV on studied genes may be dose-independent.

## Discussion

Increasing intestinal barrier permeability and inflammatory cytokines production play significant role in developing intestinal and metabolic diseases, and gut microbiota have critical role in regulating the host immune system by various signaling pathways such as TLRs (19, 20). In present study, it has been shown that *A. muciniphila *contributes to an increase in the expression of *tlr2* and *tlr4*. In the same vein, in the Ottman’s study who investigated the effect of *A. muciniphila *on HEK-Blue cells, an increase in the expression of *tlr2* and *tlr4* has been shown (20). In the another study shown that *A. muciniphila *LPS is different from *E.coli* LPS and is not considered as a strong agonist for TLR4 (21). Additionally, it has been shown that *A. muciniphila* can increase the expression of *tlr2* through the Amuc-1100 protein not LPS in mice (22). Since the LPS of commensal and pathogenic bacteria is different, it leads the host immune system to tolerance, and these stimulations are probably as a result of intestinal immune tolerance to *A. muciniphila*. Most of commensal bacteria cause immunomodulatory by producing OMVs, furthermore participate in immune system regulation and intestine homeostasis. In present study, *A.m*EV decreased *tlr4* and *tlr2* expression. In this regard, research investigating the effect of OMV of *E.coli C25*, a commensal bacterium, on *tlr4* expression in the Caco-2 cell line revealed that its OMV also has decreased *tlr4* expression (23). In a study by Shen on OMV of *Bacteroides fragilis*, it has been shown that PSA in its OMV reacting with TLR2 and triggering the secretion of anti-inflammatory cytokines (24). 

In the intestinal medium, intestinal microbiota interacts with TJ proteins, causing the protection of the integrity, the improvement of intestinal barrier function, and the protection against pathogenic bacteria and metabolites resulting from them. In the present study, we showed that *A. muciniphila *increased TJs such as* ocldn, cldn4, zo1*_,_* zo2 *and* zo3*, and in addition *Am*EV increased the expression of *ocldn* and *zo2*. These results indicate the significant role of *A. muciniphila *and *Am*EV in increasing the expression of TJ proteins which are involved in the regulation of the assembly of TJ proteins and the stability of the intestinal barrier (25). Additionally, *E. coli strain Nissle 1917 (EcN)* has been shown that increases TJ proteins such as *cldn14 *and* zo1* expression (26, 27). The effects of *A.m*EV on mice fed HFD have been investigated in a study by Chelakkot *et al*. and it has been found that EVs of this bacterium increases the expression of TJ proteins, reduces body weight, and improves metabolic function (28). A recent study showed that *A. muciniphila *regulates TJ proteins such as *Ocldn* and *Cldn3* and increases intestinal barrier integrity by affecting on TLR2*(*22*)*. In recent studies, it has been shown that the oral gavage of *A. muciniphila *can increase the thickness of the mucus and decrease the permeability of the intestinal epithelial barrier. Therefore, it can be concluded that as the thickness of the intestinal epithelial mucus decreases and the permeability of the epithelial barrier increases in the obesity, *A. muciniphila *is directly effective in controlling and reducing obesity (2). Additionally, in a study conducted on OMV of *EcN *bacterium in the intestinal cell line, it has been shown that it improves the up-regulation of TJ proteins such as *cldn14 *and *zo1* and decreases the expression of *cldn2*. Thus, it reduces the permeability of the intestinal barrier (25). There have been many studies on the safety of probiotics. The leaky gut hypothesis (the increase in the permeability of the intestinal barrier) occurs in metabolic and inflammatory bowel diseases, leading to the passage of bacteria, metabolites and their derivatives outside the intestine and the stimulation of the immune system. Considering the results obtained in this study on human intestine cell line and the significant role of *A. muciniphila *and *A.m*EV in reducing permeability and inflammation, it can be suggested that EVs of this bacterium is a more suitable treatment target than the live bacteria and can be used in the treatment of many inflammatory diseases to regulate the integrity of the intestinal barrier and reduce inflammation. Further investigations are pivotal to accredit these conclusions.
